# Gut microbiota-derived metabolites modulate Treg/Th17 balance: novel therapeutic targets in autoimmune diseases

**DOI:** 10.3389/fimmu.2025.1710733

**Published:** 2025-11-10

**Authors:** Guolin Li, Yu Xiong, Zhimin Li, Qin Yu, Shiran Li, Jingxian Xie, Siyu Zeng, Dongke Yu, Yong Yang, Jiangping Yu

**Affiliations:** 1 Department of Pharmacy, Mianyang Central Hospital, School of Medicine, University of Electronic Science and Technology of China, Mianyang, China; 2 Department of Pharmacy, Personalized Drug Research and Therapy Key Laboratory of Sichuan Province, Sichuan Provincial People’s Hospital, School of Medicine, University of Electronic Science and Technology of China, Chengdu, China; 3 School of Pharmacy, Southwest Medical University, Luzhou, Sichuan, China

**Keywords:** gut microbiota, microbial metabolites, Treg/Th17 balance, autoimmune diseases, immune regulation, therapeutic targets

## Abstract

Dysregulation of the homeostasis between regulatory T cell (Treg) and T helper 17 cell (Th17) is increasingly recognized as a pivotal mechanism in the pathogenesis of autoimmune diseases. Emerging evidence indicates that gut microbiota-derived metabolites, including short-chain fatty acids, secondary bile acids, and aromatic metabolites, modulate Treg/Th17 balance by shaping immune cell differentiation and function, thereby revealing novel therapeutic opportunities. This Review synthesizes recent clinical and preclinical findings on the influence of microbial communities and their metabolites on Treg/Th17 dynamics and examines the underlying mechanisms in representative autoimmune disorders, such as rheumatoid arthritis, systemic lupus erythematosus, Graves’ disease, autoimmune hepatitis, and myasthenia gravis. We critically evaluate current microbiome-targeted interventions and discuss their translational potential, highlighting both promises and challenges. Finally, we outline priorities for future research, focusing on multi-omic integration, the development of individualized therapeutic strategies, and rigorous clinical evaluation, to facilitate the development of safe and effective microbiota-based therapies for autoimmune diseases.

## 1 Introduction

Immune-cell imbalance — particularly the functional opposition and dysregulation between regulatory T cells (Treg) and T helper 17 cell (Th17) — has been widely recognized as a central driver of autoimmune disease pathogenesis (1, 2). Tregs are indispensable for maintaining immune homeostasis by suppressing excessive immune responses and preventing autoimmunity (3). In contrast, Th17 promote chronic inflammation and the development of autoimmune disorders through secretion of pro-inflammatory cytokines (4). Therefore, disruption of the Treg/Th17 equilibrium frequently precipitates the onset and progression of autoimmune disease (5, 6). Indeed, reduced Treg function accompanied by elevated Th17 activation is commonly observed in conditions such as rheumatoid arthritis (RA) and systemic lupus erythematosus (SLE) (7, 8).

The gut microbiota functions as a major regulator of host immunity, and microbial metabolites have emerged as key modulators of immune-cell differentiation and function (9). Accumulating clinical and preclinical studies indicate that gut microbes and their metabolic products influence autoimmune pathogenesis in part by shaping the Treg/Th17 balance (10). For example, microbial metabolites such as short-chain fatty acids (SCFAs) have been reported to promote Treg differentiation while inhibiting Th17 activation (11, 12). Such regulatory mechanisms suggest novel therapeutic avenues for autoimmune disease.

Multiple studies further indicate that loss of microbial diversity and altered metabolite abundance correlate inversely with disease activity, and that restoring microbiome composition can rebalance Treg and Th17 populations and ameliorate disease severity (8, 13). Interventions targeting the gut microbiome — including probiotic administration, dietary modification, and fecal microbiota transplantation (FMT) — have demonstrated potential to modulate immune responses and improve clinical outcomes in various settings (14, 15).

Identifying microbiota-derived metabolites that regulate immune homeostasis therefore provides new perspectives for therapeutic development and for prioritizing translational research. By synthesizing mechanistic insights and clinical evidence, this Review aims to delineate how gut microbial metabolites govern the Treg/Th17 axis, summarize disease-specific findings, and evaluate translational pathways toward microbiome-based therapies for autoimmune disease.

## 2 Interactions between gut microbiota and the immune system

### 2.1 Immunological functions of Treg and Th17 and mechanisms that maintain their balance

Treg and Th17 represent two principal CD4^+^ T-cell subsets that play opposing yet complementary roles in immune homeostasis and the regulation of immune responses (16). Treg cells restrain excessive immunity and maintain tolerance largely through the production of anti-inflammatory cytokines such as IL-10 and transforming growth TGF-β, thereby preventing autoimmune pathology (17). Numerous studies have documented quantitative and functional alterations in Treg populations across autoimmune disorders; impaired Treg function correlates closely with disease exacerbation in conditions such as RA and SLE (18, 19).

By contrast, Th17 promote inflammatory responses and contribute to autoimmune pathogenesis via secretion of pro-inflammatory cytokines including IL-17 and IL-22 (20). Although Th17 cells are important for host defense against certain pathogens, their aberrant activation can drive tissue injury and autoimmunity (21). In many autoimmune diseases, the frequency or activity of Th17 is elevated while Treg numbers or suppressive capacity are reduced, producing a net shift toward inflammation and tissue damage (22, 23).

The lineage specification of Treg and Th17 is governed in part by the interplay between key transcriptional regulators: RORγt, the lineage-defining factor for Th17, and Foxp3, the master regulator of Treg identity and function (24). The balance between RORγt and Foxp3 is critical for immune equilibrium, and disruption of their reciprocal regulation can precipitate immune dysregulation and disease (25, 26).

In autoimmune settings, the Treg/Th17 balance is disrupted. For instance, RA patients exhibit elevated Th17 cells and often defective Treg function, leading to IL-17–mediated synovitis and osteoclast activation (27). Similarly, SLE patients show increased circulating Th17 frequency correlating with disease activity (28). These examples underscore that an overactive Th17 response concurrent with impaired Treg regulation underlies pathology in RA, SLE and other autoimmune diseases.

A range of extrinsic and intrinsic cues — including environmental signals, cytokine milieus and metabolic pathways — shape the differentiation trajectories of Treg versus Th17 (29). For example, cytokines such as IL-6 and IL-23 favor Th17 differentiation, whereas TGF-β promotes Treg induction under certain contexts (30). This dynamic interconversion and competitive differentiation between the two lineages underlies both immune tolerance and pathogenic inflammation, making restoration of the Treg/Th17 balance a promising strategy to modulate immune responses and treat autoimmune disease (31, 32).

### 2.2 Regulatory effects of the gut microbiota on the Treg/Th17 balance

**Figure 1:**
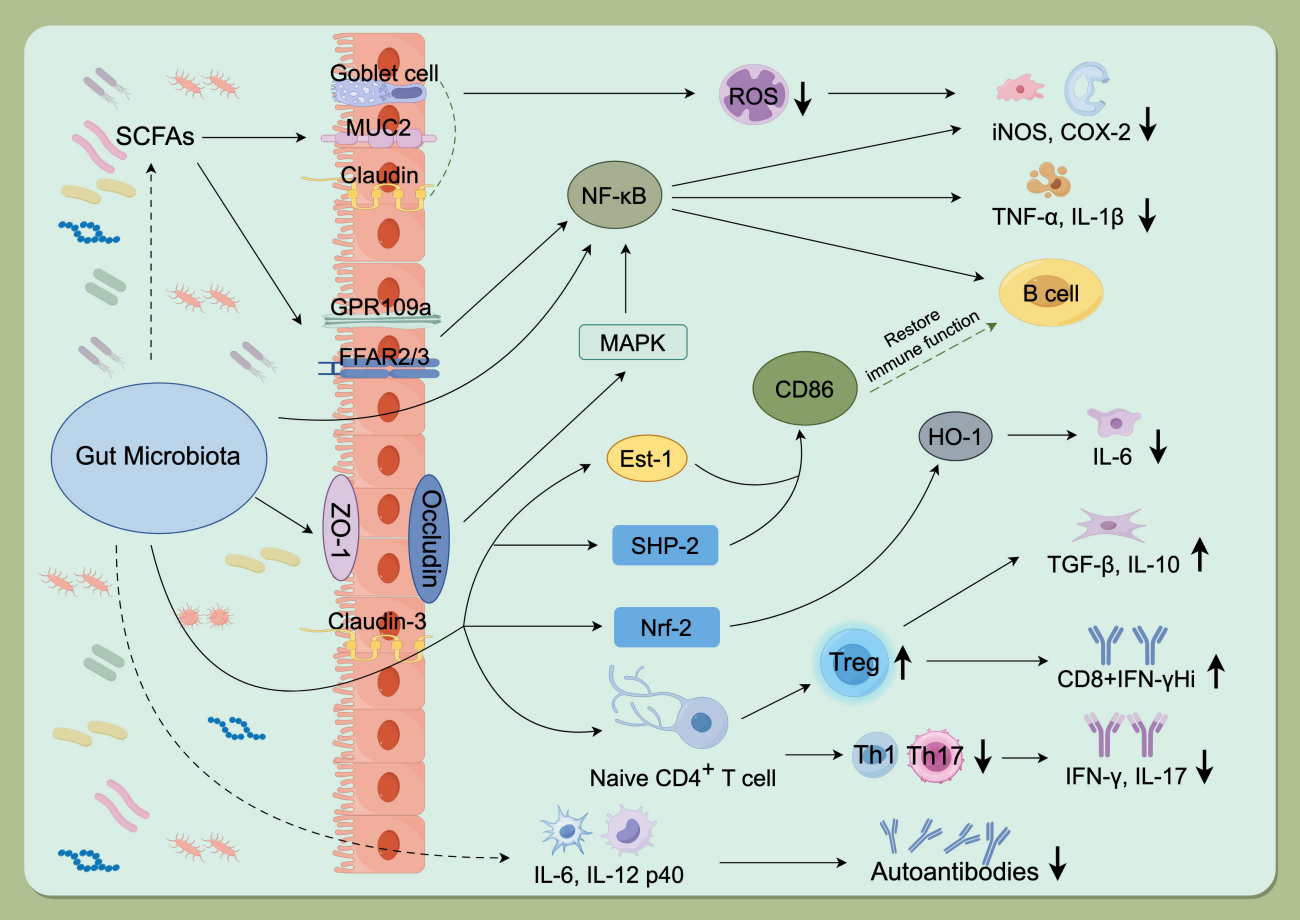
Mechanisms of gut microbiota involvement in autoimmune diseases. The gut microbiota promotes autoimmune pathogenesis by modulating key signaling pathways (NF-κB, Nrf-2), disrupting the Treg/Th1/Th17 balance, regulating the release of inflammatory factors, and producing microbial metabolites such as SCFAs. GPR109a, G protein-coupled receptor 109a; FFAR2/3, Recombinant Free Fatty Acid Receptor 2/3; SCFAs, Short chain fatty acids; MUC2, Recombinant Mucin 2; ROS, Reactive oxygen species; NF-kB, Nuclear factor kappa-B; COX-2, Cyclooxygenase 2; iNOS, Inducible nitric oxide synthase; ZO-1, Zona Occludens 1; MAPK, Mitogen-activated protein kinase; IL-1b, Interleukin-1 beta; TNF-a, Tumor necrosis factor alpha; Est-1, Estrogen sulfotransferase-1; SHP-2, SH2 domain-containing protein-tyrosine phosphatase-2; Nrf-2, NF-E2-related factor 2; HO-1, Recombinant Heme Oxygenase 1; IL-6, Interleukin-6; IL-10, Interleukin-10; TGF-b, Transforming growth factor beta; IFN-g, Interferon gamma; IL-17, Interleukin-17; IL-21/IL-22, Interleukin-21/Interleukin-22; IL-12 p40, Interleukin12 p40. (By Figdraw, ID: TWIOIab0ee).

The gut microbiota exerts profound influences on host immunity, particularly by shaping T-cell differentiation. Evidence indicates that microbial communities modulate the equilibrium between Treg and Th17 through diverse mechanisms, including the production of metabolites, antigen presentation, and regulation of mucosal immunity (33). Treg and Th17 are two major CD4^+^ T-cell subsets with opposing functions in immune regulation: Th17 drive inflammatory responses, whereas Treg cells suppress excessive immunity (34). Microbial metabolites such as SCFAs promote Treg differentiation while limiting Th17 proliferation, thereby sustaining both intestinal and systemic immune homeostasis (8). For example, Bacteroides fragilis polysaccharide Asignals via TLR2 to induce IL-10–producing Foxp3^+ Tregs (35), and segmented filamentous bacteria drive Th17 differentiation through antigen presentation and local DC-mediated cues (36). Collectively, these findings demonstrate that defined microbial species and their metabolites regulate Treg/Th17 polarization and cytokine production, maintaining immune balance within this axis (**Figure 1**) (33).

Gut microbiota dysbiosis refers to an imbalance in gut microbial ecology that disrupts health. Formally, gut dysbiosis is defined as increased pathogens and pathobionts, reduced beneficial keystone taxa, and loss of overall diversity (37). Hallmarks include loss of SCFA-producing *Firmicutes* and bloom of *Proteobacteria/enterobacteria* (38). This is often accompanied by altered microbial metabolism: e.g. reduced butyrate synthesis and depleted anti-inflammatory metabolites. In elderly or diseased individuals, dysbiosis is manifested by narrowed richness and expansion of opportunistic bacteria (39). Functionally, dysbiosis predisposes to barrier dysfunction and inflammation: irreversible microbiome shifts associate with gut barrier breakdown and systemic disease (IBD, diabetes, etc.) (40, 41). Thus, we define dysbiosis as a loss of the “healthy” core microbiota (high diversity, stable SCFA producers) coupled with metabolite derangements. In this state, local and systemic immune tolerance is undermined.).

A healthy gut microbiota can maintain the organism’s normal tolerance environment. Commensals induce intestinal DCs to produce retinoic acid and TGFβ, promoting peripheral Tregs (42). Dysbiosis increases epithelial permeability and translocation of microbial products (LPS, flagellin), triggering DC activation and IL-6/IL-23 production that favor Th17 differentiation (43). Indeed, irreversible dysbiotic changes associate with gut barrier defects and systemic inflammation (44, 45). Restoring commensal-derived signals (e.g. mucosal IgA, epithelial IL-10) is thus crucial to re-establish Treg-mediated tolerance (46, 47).

The gut ecosystem is shaped by numerous intrinsic and extrinsic factors, which in turn influence the Treg/Th17 balance. Intrinsic factors include age, sex, and genetics. For example, normal gut diversity increases during early life and plateaus in adulthood (48), but old age is associated with loss of microbial richness and depletion of SCFA-producers (49), possibly contributing to “inflammaging”. Host sex hormones also modulate the microbiota: in NOD mice, gut microbes elevated testosterone in males, protecting against autoimmunity, and transfer of male microbiota to females increased their testosterone and reduced disease (50). Such “microgenderome” effects likely contribute to the higher prevalence of autoimmune disorders in women, although the exact microbial taxa involved are still under study.

Extrinsic factors are major drivers of microbiota composition. Diet is paramount: cohorts across continents show that Western diets (high fat/sugar, low fiber) select for low-diversity communities, whereas rural or high-fiber diets support diverse, fiber-fermenting microbiota (51). For instance, a high-fat diet consistently increases the Firmicutes/Bacteroidetes ratio and reduces beneficial taxa (52). Changes can be rapid; immigrants adopt the gut microbiome of the host country within weeks of diet change (53). Conversely, lifelong Western diets can irreversibly erase key taxa (54). Geography and lifestyle also matter, stool surveys show distinct microbial signatures in Malawian and Amerindian children versus US children (51), reflecting diet, sanitation, and cultural habits. Antibiotics and drugs profoundly perturb the microbiota, even short courses cause sustained loss of diversity and expansion of resistant organisms (55).

Targeted microbial interventions — including probiotics, prebiotics, and FMT — have been shown to restore Treg/Th17 homeostasis and ameliorate immune-mediated disorders. For instance, plant-derived compounds such as phytosterols enhance SCFAs production, thereby promoting Treg differentiation, suppressing Th17 expansion, rebalancing gut microbial ecology, and attenuating inflammation (56).

Notably, the immunomodulatory influence of the gut microbiota extends beyond the intestinal tract to peripheral compartments such as the blood and spleen (33). This cross-compartment regulation underscores that microbial health impacts not only gastrointestinal physiology but also systemic immune homeostasis. Taken together, these insights highlight the substantial therapeutic potential of microbiota-based interventions in autoimmune disease, particularly through re-establishing the Treg/Th17 balance.

## 3 Molecular mechanisms by which gut microbial metabolites regulate the Treg/Th17 balance

### 3.1 SCFAs

**Figure 2:**
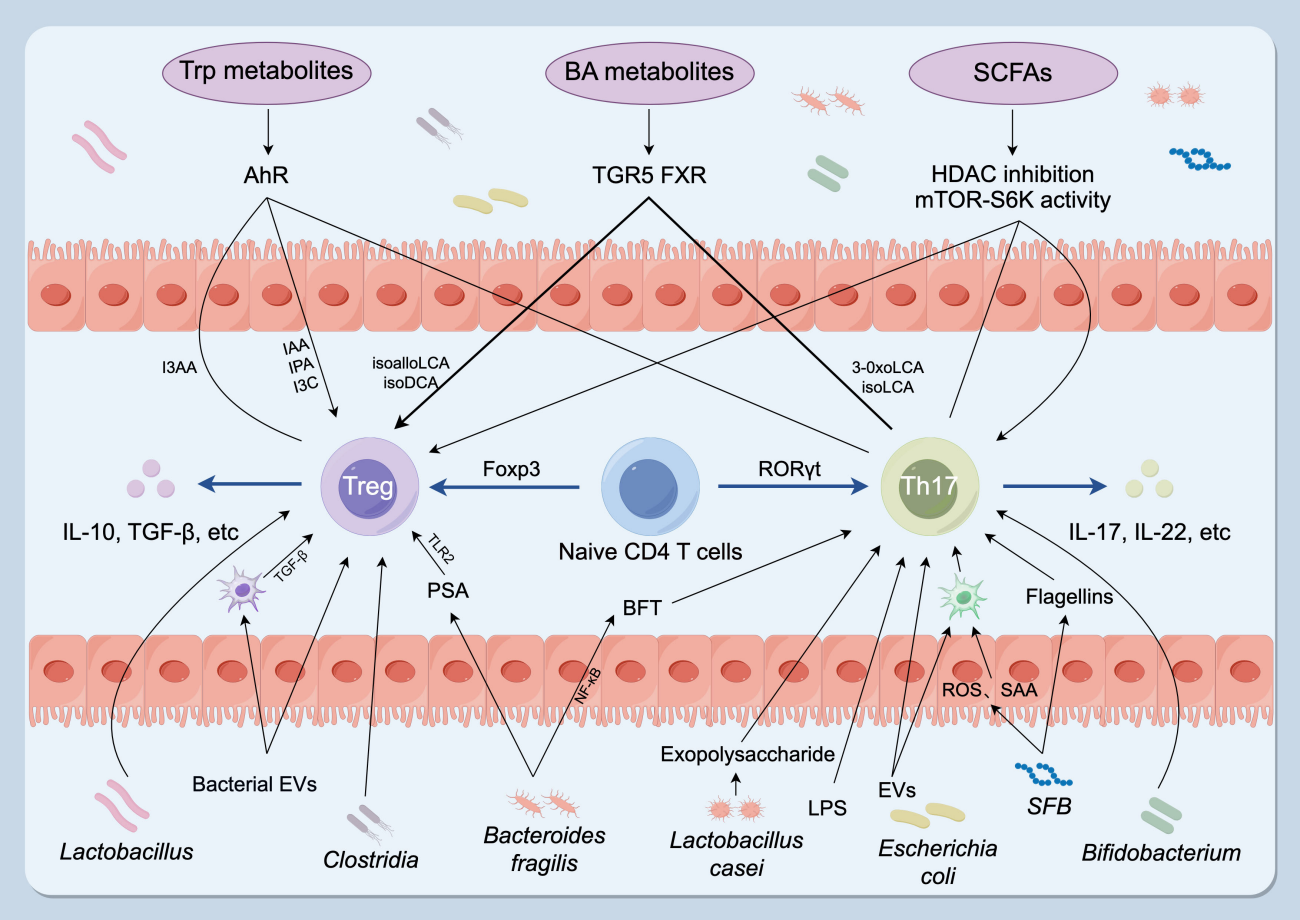
Gut microbiota and microbial metabolites regulate the Th17–Treg balance. Specific taxa influence T-cell fate: *Clostridium*, *Bacteroides fragilis* and *Lactobacillus* promote Treg development and function, whereas SFB, *Bacteroides fragilis*, *Lactobacillus* and *Bifidobacterium* stimulate Th17 differentiation and cytokine production. Microbial metabolites are key effectors: SCFAs (acetate, propionate, butyrate) act mainly via HDAC inhibition and modulation of the mTOR–S6K pathway; BA derivatives signal through TGR5 and FXR to promote Treg differentiation and/or suppress Th17 polarization; Trp-derived ligands (IAA, IPA, I3C) function as AhR agonists that favor Treg generation and restrain Th17 responses, whereas I3AA may antagonize AhR and impair Treg function. Microbial components and secreted factors — including LPS, flagellin (from SFB), exopolysaccharides (from *Lactobacillus casei*), EVs and BFT — promote Th17 differentiation. Conversely, EVs, PSA (from *Bacteroides fragilis*) and cell-surface β-glucan/galactan polysaccharides enhance Treg activity. Treg, regulatory T cell; Th17, T helper 17 cell; SFB, segmented filamentous bacteria; SCFAs, short-chain fatty acids; BA, bile acid; Trp, tryptophan; HDACs, histone deacetylases; TGR5, G-protein-coupled bile acid receptor; FXR, farnesoid X receptor; IAA, indole-3-acetic acid; IPA, indole-3-propionic acid; I3C, indole-3-carbinol; AhR, aryl hydrocarbon receptor; I3AA, indole-3-acetaldehyde; LPS, lipopolysaccharide; EVs, bacterial extracellular vesicles; BFT, *Bacteroides fragilis* toxin; PSA, polysaccharide A. (By Figdraw, ID: ATTYS1c1c0).

Short-chain fatty acids (SCFAs), principally acetate, propionate, and butyrate, are microbial metabolites generated through fermentation of dietary fibers in the gut (57). Accumulating evidence highlights their indispensable roles in intestinal health and immune regulation. The study found that SCFAs enhance the stability of Treg cells and suppress the activity of Th17 cells, thereby contributing to the maintenance of immune system homeostasis (58). SCFAs, particularly butyrate, function as a primary energy source for colonocytes. Simultaneously, they modulate host immunity by promoting regulatory T-cell differentiation and mucosal tolerance through mechanisms that include GPR43-mediated signaling and epigenetic regulation via histone deacetylase inhibition (59). One key pathway is engagement of SCFA-sensing G protein–coupled receptors: microbial acetate, propionate and butyrate activate FFAR2 (GPR43) and FFAR3 (GPR41) on mucosal cells and leukocytes. FFAR2 signaling is necessary for SCFA-driven expansion of colonic Foxp3^+ regulatory T cells and protection from T-cell-transfer colitis, while SCFAs additionally act via HDAC inhibition and mTOR–S6K modulation to stabilize Foxp3 expression and limit pro-inflammatory Th17 responses (**Figure 2**) (60). For instance, propionate activates the GPR43–cAMP/PKA–CREB signaling cascade, leading to expansion of Treg cells and increased secretion of IL-10 and TGF-β (8). In animal models of autoimmune disease such as RA, SCFAs supplementation significantly alleviates joint inflammation and tissue injury.

SCFAs also regulate immune function through inhibition of histone deacetylases (HDACs), thereby stabilizing the Treg phenotype (61). Butyrate, for example, promotes Foxp3 expression via HDAC inhibition, strengthening Treg suppressive capacity. This epigenetic mechanism contributes to the maintenance of tolerance and prevents excessive immune activation, thereby curbing autoimmune responses (62).

Moreover, SCFAs reinforce tolerance by balancing pro- and anti-inflammatory cytokine production. They downregulate pro-inflammatory mediators such as IL-6 and IL-23 while enhancing IL-10 and TGF-β expression, a shift repeatedly observed across models of autoimmune disease (63, 64). These findings suggest that SCFAs hold substantial translational promise as immunoregulatory metabolites.

*In vivo* studies support this potential: supplementation with SCFAs attenuates clinical symptoms in models of RA and inflammatory bowel disease, while improving barrier integrity and reducing systemic inflammation (65). Taken together, SCFAs emerge as key microbial products that modulate the Treg/Th17 axis through convergent signaling and epigenetic pathways, thereby promoting tolerance and constraining autoimmune pathology. Their capacity to re-establish immune balance positions SCFAs as both mechanistic targets and therapeutic candidates for autoimmune disease (66, 67).

### 3.2 Secondary bile acids

The gut microbiota converts primary bile acids into secondary bile acids through a series of complex metabolic processes, exerting profound effects on the host immune system. Primary bile acids are synthesized in the liver and secreted into the intestine via bile, where microbial enzymatic reactions transform them into secondary bile acids such as deoxycholic acid (DCA) and lithocholic acid (LCA) (68, 69). These metabolites not only contribute to the composition of the bile acid pool but also regulate host metabolism and immune responses by binding to bile acid receptors expressed on host cells. Accumulating evidence indicates that the synthesis of secondary bile acid is tightly linked to the composition and functional state of the gut microbiota, with microbial diversity significantly shaping this metabolic process (70, 71). Moreover, secondary bile acids play crucial roles in maintaining intestinal barrier integrity, modulating immune responses, and mediating anti-inflammatory effects.

The immunomodulatory properties of secondary bile acids are particularly evident in the regulation of Treg and Th17 (72). FOXP3^+^ Tregs are key mediators of immune tolerance, and their dysfunction or depletion is closely associated with the pathogenesis of multiple autoimmune diseases (73). Studies have shown that secondary bile acids, particularly DCA and LCA, promote the differentiation of FOXP3^+^ Treg while suppressing Th17 cell development through specific signaling pathways (72, 74). In autoimmune disorders such as multiple sclerosis (MS), a deficiency of secondary bile acids correlates with reduced Treg abundance and increased Th17 responses, suggesting a critical role of bile acid metabolites in regulating CNS autoimmunity (75). Modulating gut microbial composition to enhance secondary bile acid production may therefore represent a novel therapeutic strategy for MS and related diseases.

In MS patients, the production of secondary bile acids is markedly reduced compared with healthy controls, paralleling impaired immune regulation. Specifically, microbial taxa responsible for secondary bile acid biosynthesis are diminished, leading to decreased intestinal levels of immunoregulatory metabolites such as DCA and LCA (72). This reduction compromises Treg function while enhancing Th17 differentiation, driving CNS inflammation and disease progression. Therapeutic strategies aimed at restoring bile acid balance have shown promise: supplementation with DCA or LCA in experimental MS models effectively restored immune homeostasis, reduced Th17 frequency, promoted Treg differentiation, and alleviated clinical symptoms (72, 74). These findings not only highlight the therapeutic potential of secondary bile acid supplementation but also deepen our understanding of microbiota–immune system crosstalk. Targeting bile acid metabolism may thus offer new treatment options for MS, improving both immune balance and patient outcomes.

### 3.3 Tryptophan and other aromatic metabolites

Tryptophan (Trp), an essential aromatic amino acid in humans, has emerged as a critical substrate for generating immunoregulatory metabolites such as indole and indole-3-propionic acid. Increasing evidence indicates that Trp-derived indole compounds modulate the balance between Treg and Th17 primarily through binding to the aryl hydrocarbon receptor (AhR) (58). AhR is a ligand-activated transcription factor that governs diverse physiological processes, including immune regulation. Trp metabolism therefore not only shapes the local intestinal immune microenvironment but also exerts systemic immunomodulatory effects by directing T-cell differentiation and function (76). In murine models, indole compounds have been shown to promote Treg generation while suppressing Th17 differentiation, thereby sustaining immune tolerance and dampening inflammatory responses (77). These findings highlight the therapeutic potential of targeting Trp metabolism to reestablish Treg/Th17 homeostasis in autoimmune diseases.

Trp metabolism is a key microbiota–immune interface. Both host enzymes (IDO1/IDO2, TDO) and bacterial pathways convert Trp into bioactive compounds (9, 78). The host IDO-mediated kynurenine (Kyn) pathway generates metabolites that modulate T cells (79). Microbiota-derived indole and indole-derivatives (e.g. indole-3-aldehyde, indole propionic acid) act via AhR on immune cells and mucosal cells (80). These Trp metabolites generally promote Tregs and suppress Th17. For instance, Kyn and 3-HAA synergistically drive naïve CD4^+ T cells to Foxp3^+ Tregs in the presence of DCs (81). Indole derivatives from commensals activate AhR to induce IL-22 and IL-10, enhancing barrier function and Treg stability. Notably, RA patients show perturbations in Trp metabolism: serum kynurenic and xanthurenic acids (Treg-promoting) are decreased, while neurotoxic quinolinic acid is elevated (82). Experimental arthritis was improved by supplementing enzyme activities to boost Kyn metabolites. In SLE, increased IDO activity and high Kyn/Trp ratio correlate with disease and fatigue (83). These data link dysbiotic Trp metabolism to Th17/Treg imbalance in autoimmunity. Overall, Trp-derived metabolites constitute another axis by which gut microbiota shape T cell fate.

## 4 Advances in the study of gut microbial metabolites and Treg/Th17 balance in representative autoimmune diseases

### 4.1 Rheumatoid arthritis

Rheumatoid arthritis (RA) is a chronic autoimmune disorder in which mounting evidence links the gut microbiome to disease onset and progression. Patients with RA frequently exhibit reduced microbial diversity, diminished levels of SCFAs, and an increased proportion of Th17, collectively contributing to aberrant immune activation and persistent inflammation (84, 85). Dysbiosis disrupts the delicate equilibrium between Treg and Th17, thereby fueling chronic inflammatory responses (8, 86). **Table 1** summarizes mechanistic and clinical evidence for gut microbiota–derived metabolites in modulating the Treg/Th17 axis across representative autoimmune diseases.

**Table 1:** Microbial metabolite evidence for Treg/Th17 modulation in autoimmune disease.

Disease	Representative metabolite	Sample/Model	Effect on Treg/Th17	Main finding	References
RA	Butyrate (SerBut)	Mouse models	↑ Treg; ↓ Th17/reduced inflammatory readouts	SerBut improved oral bioavailability and markedly reduced arthritis and neuroinflammation in murine models without overt systemic immunosuppression.	(87)
	Butyrate (SCFAs)	Animal and human observational studies	Mechanistic evidence supports promotion of Foxp3+ Treg and limitation of Th17	RA patients exhibit reduced butyrate-producing bacteria; perturbation correlates with barrier dysfunction and systemic inflammation.	(88)
	Butyrate (micelle/prodrug delivery)	CAIA arthritis mouse model	↑ Treg; ↓ inflammatory mediators	Targeted delivery produced long-lasting immunomodulatory effects and suppressed arthritis after limited dosing.	(89)
MS	Butyrate (SerBut)	EAE mouse model	↑ Treg; ↓ Th17 in CNS/peripheral compartments	SerBut suppressed neuroinflammation in EAE model and reduced clinical scores.	(87)
	Methyl butyrate (SCFAs derivative)	EAE mouse model	↓ Th17; ↑ regulatory markers (Treg increase reported)	Methyl butyrate administration reduced clinical severity and improved histopathology in EAE.	(90)
	FMT (microbiota intervention)	Small randomized pilot trial in RRMS patients	Pilot biomarkers suggested shifts consistent with enhanced mucosal tolerance (potential ↑ Treg/↓ Th17)	FMT was safe/tolerable; improvements in intestinal permeability and microbiota composition observed; biomarker changes suggested immunomodulatory effects.	(91)
	Bile-acid derivatives	Human isolate screen, mechanistic ex vivo assays, cohort associations	↓ Th17 differentiation; promotes regulatory milieu	Identified human bacteria producing bile-acid derivatives that inhibit Th17 differentiation; linked metabolites to host Th17 gene expression.	(92)
SLE	Tryptophan-derived indoles/AhR ligands (IPA/IAld)	Review of mechanisms and disease links	Ligand-dependent: some ligands favor Treg/IL-22; others favor Th17	Trp metabolism converges on AhR activation to modulate immune responses; ligand specificity determines Treg vs Th17 outcomes.	(93)
	Indole derivatives (AhR ligands)	Mechanistic review with animal and human examples	Context- and ligand-dependent modulation of Treg/Th17	AhR ligand identity determines Treg vs Th17 differentiation; suggests potential for selective AhR modulators.	(94)
	Altered tryptophan metabolites (kynurenine, indoles)	Human cohort metabolomics	Associations consistent with dysregulated Treg/Th17 balance	Altered Trp metabolism correlates with disease activity and immune signatures; supports targeted intervention.	(93–96)
T1D	Acetate/Butyrate (diet-released SCFAs)	NOD mice/dietary intervention models	↑ Foxp3+ Treg; ↓ Th17/reduced autoimmune destruction	Diets releasing acetate/butyrate increased SCFAs, reduced insulitis and T1D incidence; increased colonic Treg.	(97)
	Butyrate	Mechanistic murine studies	↑ Treg migration/function; ↓ diabetogenic responses	Butyrate-induced colonic Treg can migrate to pancreas and draining lymph nodes; increased Treg migration contributed to suppression of autoimmune diabetes.	(98)
	Acetate/Butyrate (after HAMSAB feeding)	Preclinical NOD data + early human pilot data	↑ Treg; ↓ inflammatory markers in models; human signals preliminary	Dietary intervention increased SCFAs and correlated with immune changes consistent with increased regulatory responses; early human studies show metabolome/immune modulation.	(99)
	Acetate (SCFAs)	NOD mice	Associated with increased regulatory signals and reduced pathogenic T cell activation	Acetate reduced gut bacteria–induced IgA and decreased insulitis severity, suggesting protective immunoregulatory effects.	(100)
AIH	Bile-acid derivatives	Mouse models and mechanistic *in vitro* assays	3-oxoLCA → ↓ Th17 (RORγt binding); isoalloLCA → ↑ Treg (Foxp3 enhancement)	3-oxoLCA inhibited Th17 differentiation via direct RORγt binding; isoalloLCA promoted Foxp3 expression and Treg differentiation via CNS3-dependent mechanism.	(100)
	3-oxoLCA; isoLCA (human bacterial producers)	Screening of human isolates; cohort association analyses	↓ Th17; supports regulatory milieu	Identified human bacteria and enzymes producing these bile-acid derivatives; metabolites associate negatively with Th17 signatures in cohorts.	(92)
	isoallolithocholic acid (isoalloLCA)	Mechanistic *in vitro* and mouse studies	↑ Treg via mitochondrial/transcriptional pathways	isoalloLCA enhances Treg differentiation and function; mechanistic evidence of bile-acid-mediated promotion of regulatory responses.	(101)

Treg, regulatory T cell; Th17, T helper 17 cell; RA, rheumatoid arthritis; SCFAs, short-chain fatty acids; CAIA, Collagen Antibody-Induced Arthritis; MS, multiple sclerosis; EAE, experimental autoimmune encephalomyelitis; SLE, systemic lupus erythematosus; AhR, aryl hydrocarbon receptor; IPA, indole-3-propionic acid; IAld, indole-3-aldehyde; T1D, type 1 diabetes; NOD, Non-Obese Diabetic; AIH, autoimmune hepatitis; FMT, fecal microbiota transplantation. "↑" represents increase; "↓" represents decrease.

Notably, SCFAs concentrations are significantly lower in RA patients compared with healthy controls (102). SCFAs not only sustain intestinal barrier integrity but also orchestrate immune regulation by promoting Treg differentiation and suppressing Th17 activation (103, 104). For instance, butyrate enhances Treg expansion and IL-10 secretion via GPR43 signaling while simultaneously attenuating Th17 polarization, underscoring its potential as a therapeutic target in RA (8, 105).

Current therapies such as methotrexate have been shown to partially restore gut microbial composition and increase SCFAs levels, thereby rebalancing Treg and Th17 populations. Clinical studies demonstrate that methotrexate treatment improves microbial diversity and structure in RA patients, accompanied by reduced inflammatory markers (106, 107). These findings suggest that targeting the gut microbiota to modulate immune responses may represent a promising adjunctive strategy for RA management.

Dysregulation of Trp metabolism has emerged as a critical link connecting the gut microbiota, host immunity, and disease activity in RA. Recent studies highlight its dual role: an imbalance in the host kynurenine pathway, characterized by decreased protective metabolites (e.g., kynurenic acid) and elevated pro-inflammatory metabolites (e.g., quinolinic acid), correlates with disease severity, and restoring this balance shows therapeutic potential in animal models (82, 108). Concurrently, gut microbiota-derived indole derivatives exert opposing effects. For instance, indole-3-propionic acid maintains immune homeostasis and alleviates arthritis by activating AhR (84), whereas unmodified indole promotes a pro-inflammatory Th17 response, exacerbating disease (109). This metabolic heterogeneity underscores that Trp metabolism is not only a source of robust biomarkers for RA but also a promising, yet complex, therapeutic target requiring precise modulation.

Probiotic interventions have also shown therapeutic promise. For example, *Lactobacillus casei* has been reported to alleviate arthritis by reshaping gut microbial communities and enhancing SCFAs production. Specifically, *Lactobacillus casei* CCFM1074 reduced Th17 cell proportions while expanding Treg populations, thereby ameliorating disease severity in RA mouse models (104, 110). Such evidence highlights the potential of probiotic supplementation as an adjunct therapy for RA, reinforcing the concept that gut microbiota modulation could be harnessed as a novel therapeutic avenue.

Collectively, these studies demonstrate that gut microbial metabolites play a pivotal role in regulating the Treg/Th17 balance in RA. Future research should aim to delineate the precise mechanisms through which microbial metabolites influence RA pathogenesis and explore their translational potential in clinical settings (111, 112).

### 4.2 Systemic lupus erythematosus

In systemic lupus erythematosus (SLE), growing attention has been directed toward the regulatory effects of gut microbiota and their metabolites on immune homeostasis. SLE is a complex autoimmune disease characterized by an imbalance between Treg and Th17, leading to chronic inflammation and multi-organ damage (113). Experimental studies have revealed that specific microbial taxa are markedly reduced in SLE models, whereas supplementation with these key strains can effectively restore the Treg/Th17 balance and ameliorate disease severity (114).

In the MRL/lpr mouse model, species such as *Eisenbergiella massiliensis*, *Lacrimispora saccharolytica*, and *Hungatella xylanolytica* were significantly decreased following disease onset. Their depletion was closely associated with reduced levels of metabolites including 5-cholestenol, cholesterol, p-cresol, and indole (114). These metabolites are linked to immune regulation and may influence the Treg/Th17 axis. Restoration of these microbial populations was shown to improve SLE symptoms and contribute to re-establishing Treg/Th17 balance (114).

Supplementation with *Bifidobacterium* has also demonstrated beneficial effects in both clinical and preclinical studies. Multiple investigations have confirmed that the abundance of *Bifidobacterium* is significantly reduced in SLE patients, correlating with impaired Treg function and increased Th17 activity (10). Accordingly, *Bifidobacterium* and its metabolites not only correct gut dysbiosis but also restore Treg-mediated suppression, thereby reducing Th17-driven inflammation, alleviating clinical symptoms, and mitigating renal injury in SLE (115).

SCFAs, key metabolites derived from microbial fermentation of dietary fibers, also play a pivotal role in regulating the Treg/Th17 axis (116). SCFAs production is essential for gut health and modulates immune cell differentiation and function to restrain autoimmune responses (62). In SLE models, SCFAs deficiency has been directly linked to increased Th17 activity and Treg dysfunction (117). In addition, lipopolysaccharide (LPS), a structural component of Gram-negative bacteria, exerts dual immunological effects: at low concentrations it stimulates appropriate immune activation, whereas at high concentrations it triggers excessive inflammation and immune dysregulation (118). In SLE and other autoimmune diseases, disruption in the balance of SCFAs and LPS has been implicated in disease pathogenesis (119). Hence, deciphering the roles of these microbial products provides an important foundation for developing novel immunotherapies.

### 4.3 Graves’ disease

In patients with Graves’ disease (GD), gut microbiota dysbiosis has been identified as a key contributing factor. Studies have demonstrated that supplementation with *Bacteroides fragilis* and its metabolite propionate can effectively modulate the Th17/Treg ratio, thereby attenuating inflammatory responses and improving immune homeostasis (120). Specifically, experimental evidence indicates that oral administration of *Bacteroides fragilis* or propionate markedly reduced levels of pro-inflammatory cytokines, total thyroxine, and thyrotropin receptor antibodies in GD mouse models, while simultaneously decreasing the proportion of circulating Th17 and increasing the frequency of Treg (121). These immunological shifts not only alleviated systemic inflammation but also ameliorated hyperthyroid symptoms and diminished the autoimmune response against thyroid-stimulating hormone receptor.

Moreover, *Bacteroides fragilis* and propionate significantly reduced pro-inflammatory cytokines and the proportion of M1 macrophages in thyroid tissues, while enhancing Treg cells and M2 macrophages, collectively mitigating thyroid inflammation and hypertrophy. These findings highlight the pivotal role of gut microbes and their metabolites in GD pathogenesis and underscore their therapeutic potential.

Importantly, combining *Bacteroides fragilis* or propionate with the conventional drug methimazole significantly improved pathological changes in GD mice and allowed for a reduced methimazole dosage (121). This synergistic effect not only enhanced therapeutic efficacy but also minimized adverse drug reactions, providing a rationale for microbiota-based therapies as adjuncts to standard regimens. Such strategies may represent safer and more effective treatment paradigms for GD.

Additionally, analysis of the gut microbiota in 162 patients with mild and severe GD, compared with healthy controls, revealed significant taxonomic and functional alterations (122). These distinct microbial signatures hold promise as non-invasive diagnostic biomarkers for GD and provide a foundation for future clinical applications.

### 4.4 Autoimmune hepatitis

Autoimmune hepatitis (AIH) is a chronic, immune-mediated liver disease characterized by the breakdown of immune tolerance and aberrant immune attacks against hepatic autoantigens (123). Increasing evidence indicates that gut microbiota and their metabolites play pivotal roles in the pathogenesis of AIH. Patients with AIH commonly exhibit gut dysbiosis, with markedly reduced microbial diversity compared with healthy individuals (124). Such alterations not only reshape the intestinal immune milieu but may also influence hepatic immunity via the gut–liver axis.

Changes in gut microbial composition have been shown to directly affect T cell function, particularly the balance between Treg and Th17 (125). Tregs are critical for maintaining immune tolerance and suppressing autoimmunity, whereas Th17 cells are strongly associated with pro-inflammatory responses. In AIH models, disruption of the Treg/Th17 balance exacerbates hepatic injury (126). For example, dysregulation of Trp metabolism can impair SCFAs production, resulting in diminished Treg function and enhanced Th17 activation (127).

Modulating gut microbiota composition and its metabolites has emerged as a promising strategy to restore Treg/Th17 homeostasis and attenuate liver injury. Studies have demonstrated that supplementation with specific probiotics enhances Treg proportions while suppressing Th17 activity in AIH mouse models (128, 129). For instance, *Bifidobacterium animalis* ssp. *lactis* has shown therapeutic potential by strengthening intestinal barrier integrity and modulating hepatic immune cell responses (130).

Moreover, metabolites such as SCFAs possess potent immunomodulatory properties, promoting Treg differentiation while inhibiting Th17 activation (127). In AIH, restoration of a healthy gut microbiota not only improves systemic immune regulation but also mitigates hepatic inflammation and tissue damage. By targeting gut microbial communities and their metabolic pathways to re-establish Treg/Th17 equilibrium, significant hepatoprotective effects can be achieved, offering novel therapeutic targets and translational strategies for AIH management (131, 132). Thus, interventions focused on gut microbes and their metabolites represent a promising avenue for AIH therapy.

### 4.5 Myasthenia Gravis

Myasthenia Gravis (MG) is an acquired neuromuscular autoimmune disorder characterized by impaired signal transmission at the neuromuscular junction, resulting in muscle weakness and fatigability (133). Recent studies have implicated gut microbial dysbiosis in the onset and progression of MG, with evidence pointing to a concurrent functional imbalance between Treg and Th17 as a key pathogenic mechanism (134).

Compared with healthy controls, patients with MG exhibit reduced gut microbial diversity and abundance, notably a depletion of bacterial taxa that produce SCFAs, which is considered a contributor to immune dysregulation (135). For example, marked decreases in beneficial genera such as *Faecalibacterium* have been directly associated with impaired Treg function, a deficit that may permit excessive activation of Th17 and thereby exacerbate the pathological cascade of MG (136). These observations suggest that restoring microbial balance could ameliorate symptoms by re-establishing the equilibrium between Treg and Th17 and dampening autoreactive immune responses.

Modulating the gut microbiota and its metabolites has therefore been proposed as a novel therapeutic avenue. Several investigations report that supplementation with specific probiotics or with SCFAs improves clinical and immunological features of MG, promoting immune rebalancing (137). For instance, butyrate supplementation increases Treg numbers and suppresses Th17 activity, thereby restoring the Treg/Th17 balance and mitigating pathological manifestations in experimental models (137). In addition, certain phytochemicals such as curcumin have shown potential in MG mouse models, apparently acting via modulation of the gut microbiome and elevation of SCFA levels (138).

Together, these findings indicate that targeted interventions — including probiotic therapy and dietary or metabolite supplementation — may offer new treatment options for patients with MG. Such strategies aim not only to restore immune homeostasis but also to improve patient quality of life and reduce reliance on conventional immunosuppressants. Future clinical research should prioritize rigorous evaluation of diverse microbiome-modulating approaches to determine their safety, efficacy and translational potential in MG management.

## 5 Therapeutic translation of gut microbial metabolites in regulating Treg/Th17 balance

### 5.1 Microbial and metabolite supplementation therapies

Recent years have seen growing interest in the capacity of probiotics and their metabolites to shape the gut microbiome and modulate host immune responses. Evidence indicates that oral probiotic supplementation can ameliorate clinical features of various autoimmune disorders. For example, administration of *Limosilactobacillus reuteri* DSM 17938 was reported to improve the balance between Treg and Th17, thereby slowing autoimmune progression driven by Treg deficiency (139). SCFAs, such as butyrate, are key microbial metabolites that suppress activation of Th17 while promoting proliferation of Treg, and have been shown to reduce intestinal inflammation in murine models (33).

Supplementation with particular strains, notably members of *Bifidobacterium* and *Lactobacillus*, has produced marked benefits in preclinical and clinical studies. For instance, *Bifidobacterium* supplementation not only remodels gut community structure but also mitigates pathological changes in autoimmune hepatitis by modulating host immune responses (115).

Beyond probiotics and SCFAs, secondary bile acids and their derivatives exert important immunoregulatory effects. Secondary bile acids influence intestinal immunity and microbial ecology by engaging specific host receptors, and perturbations in bile acid pools have been linked to autoimmune disease pathogenesis via effects on barrier function and immune-cell activity (140).

FMT has emerged as a promising therapeutic modality: by transferring a healthy donor microbiome to a patient, FMT aims to restore microbial balance, improve immune function and reduce inflammatory burden. Multiple studies report significant effects of FMT in the treatment of autoimmune conditions, supporting its further investigation (141).

For example, studies in RA have shown that FMT can significantly improve clinical symptoms, which correlates with increased gut microbial diversity (142). Moreover, FMT has been found to rebalance Treg/Th17 ratios, counteracting immune dysregulation caused by gut microbiota disturbances (143).

Clinically, FMT has been applied in the treatment of several autoimmune diseases, particularly inflammatory bowel diseases such as Crohn’s disease and ulcerative colitis, with encouraging outcomes (144). By restoring microbial diversity, FMT not only alleviates gastrointestinal symptoms but also promotes systemic immune reconstitution (145). However, further research is needed to establish long-term efficacy and optimize procedural standards for clinical application.

### 5.2 Metabolite-targeted delivery systems and nanotechnology

The clinical application of microbial metabolites, which play a crucial role in modulating immune responses, is often limited by their low oral bioavailability. This is particularly true for polar small-molecule metabolites such as itaconate (IA), whose intracellular efficacy often requires high exogenous concentrations to achieve therapeutic effects (146). To address this challenge, nanotechnology-based delivery systems have been developed to enhance metabolite bioavailability by improving solubility and stability. For instance, polyester-based polymeric microparticles enable endogenous delivery of small-molecule metabolites via macrophage phagocytosis, significantly increasing their effective concentration within immune cells while exhibiting low cytotoxicity (147). Moreover, the design of nanocarriers allows for targeted delivery, enabling selective release to specific cells or tissues and maximizing therapeutic outcomes.

Stimuli-responsive release systems represent a principal application of contemporary nanotechnology, enabling precise therapeutic release under defined physiological conditions (148). Using this strategy, nanoparticle-based carriers (nanocarriers) can remain stable within the intestinal microenvironment while discharging their payloads in response to specific triggers — for example, changes in pH or temperature — thereby allowing tight control over both the gut microbiota and immune-cell function. For instance, multifunctional particles engineered by nanotechnology have been shown to adjust their release profiles according to physiological variations in the intestine, effectively reshaping local immune responses and offering new therapeutic avenues for autoimmune disease (149). Moreover, combining targeted delivery of microbial metabolites with stimuli-responsive release increases treatment selectivity and reduces systemic adverse effects, thereby strengthening the clinical potential of metabolite-based interventions.

By integrating advanced nanotechnologies with intelligent release modalities, researchers are accelerating the clinical translation of metabolite-centered therapies for autoimmune disorders; these platforms substantially improve the precision and efficacy of drug delivery and help address many of the current therapeutic challenges.

### 5.3 Dietary interventions and lifestyle modifications

High-fiber diets have garnered increasing research interest, particularly in the context of managing autoimmune diseases. Studies have shown that dietary fiber is fermented by gut microbiota to produce SCFAs — such as butyrate, propionate, and acetate — which play essential roles in regulating immune balance (150). High-fiber intake has been demonstrated to significantly improve the immune profile in mouse models of RA, particularly by modulating the balance between Th17 and Treg. For example, one study revealed that a diet rich in pectin and inulin markedly reduced the severity of collagen-induced arthritis in mice and corrected aberrant T-cell differentiation by enhancing SCFAs production, thereby ameliorating immune responses (85). SCFAs not only promote Treg expansion via activation of the GPR43 receptor but also inhibit Th17 polarization, contributing to improved autoimmune outcomes (8).

Furthermore, SCFAs are closely associated with intestinal barrier integrity. By enhancing tight junctions between intestinal epithelial cells and increasing the expression of mucin Muc2, SCFAs help maintain gut barrier function, thereby reducing systemic inflammation (151). Increased SCFAs levels have been correlated with elevated Treg frequencies and reduced populations of Th1 and Th17, further supporting their therapeutic potential in autoimmune conditions (152). Therefore, implementing high-fiber diets as an intervention strategy can not only reshape the gut microbial composition but also modulate immune responses through enhanced SCFAs production, offering a novel non-pharmacological approach to autoimmune disease management.

The design of personalized dietary regimens should take into account individual gut microbial compositions. Advances in microbiome research have revealed considerable interindividual variability in gut microbiota, suggesting that uniform dietary interventions may not be effective for all patients. Tailored nutritional strategies can more effectively improve an individual’s immune status and overall health. For instance, one study showed that different dietary compositions variably influenced gut microbiota, which in turn affected immune responses and disease progression in mice (153).

In the management of autoimmune diseases, designing diet plans based on an individual’s microbiome profile may help optimize SCFAs production. Certain individuals may respond more favorably to specific types of dietary fiber, which should be prioritized in their nutritional intake. Such personalized dietary interventions can enhance microbial diversity and modulate immune cell ratios — particularly the Treg/Th17 balance — thereby alleviating autoimmune responses (154, 155).

### 5.4 Combined pharmacotherapy strategies

In the treatment of autoimmune diseases, conventional immunosuppressants, though effective, are often associated with significant side effects and high relapse rates (156). Consequently, developing combined therapeutic strategies to enhance efficacy and reduce drug dosage has become a major research focus. Microecological modulators — particularly probiotics and their metabolites — have recently demonstrated considerable potential in modulating immune responses, offering novel avenues for the treatment of autoimmune disorders.

For instance, studies have shown that *Bacteroides fragilis* and its metabolite propionate can significantly ameliorate disease manifestations in a mouse model of GD by restoring the Treg/Th17 balance (121). Oral supplementation with *Bacteroides fragilis* effectively reduced inflammatory cytokine levels, increased the proportion of Treg, and decreased Th17 cell frequency, thereby attenuating systemic inflammation, hyperthyroidism, and autoimmune responses. More importantly, the combination of *Bacteroides fragilis* with the conventional immunosuppressant methimazole not only improved pathological outcomes but also substantially reduced the required dosage of methimazole, indicating a synergistic therapeutic effect between microecological modulators and traditional drugs (121).

Further studies support the efficacy of combining microbial modulators with immunosuppressants. In RA, for example, probiotic administration has been shown to modulate gut microbiota composition and promote the production of anti-inflammatory metabolites such as SCFAs, leading to improved immune function and alleviated disease symptoms. Clinical evidence indicates that probiotics can reduce the abundance of harmful bacteria, enhance Treg functionality, and suppress Th17 activity, ultimately contributing to effective RA management (157, 158).

Combining microbiome-directed interventions with conventional immunosuppressive agents can both potentiate therapeutic benefit and mitigate drug-related adverse effects, thereby improving patients’ quality of life. Such combination strategies open new avenues for managing autoimmune diseases; future work should continue to define the mechanisms of action of distinct microbiome modulators and determine their optimal pairings with standard drugs to enable personalized regimens and enhanced clinical efficacy.

In GD, co-administration of *Bacteroides fragilis* and methimazole has produced marked therapeutic effects. Experimental data indicate that *Bacteroides fragilis* not only attenuates disease manifestations by recalibrating immune-cell populations but also acts synergistically with methimazole to permit dose reduction and lower side-effect burden. Specifically, supplementation with *Bacteroides fragilis* significantly decreased serum levels of inflammatory mediators — including proinflammatory cytokines and thyroid-associated autoantibodies — while increasing the frequency of Treg and suppressing the activity of Th17, thereby reducing systemic inflammation and the degree of thyrotoxicosis (121).

The mechanistic basis for this synergy likely involves microbiota-driven restoration of intestinal ecology and enhanced production of SCFAs, metabolites that play important roles in shaping host immune responses. In addition, combined treatment with *Bacteroides fragilis* and methimazole has been shown to improve histopathological features and to allow clinically meaningful reductions in methimazole dosing, suggesting that adjunctive microbiome therapy can preserve efficacy while improving tolerability (121).

Future studies should evaluate the efficacy of *Bacteroides fragilis* or other microbial modulators in combination with a broader range of immunosuppressants, and systematically assess the impact of dose, timing and route of administration on therapeutic outcomes. In summary, integrating microbiome-directed agents with standard pharmacotherapy offers a promising strategy for autoimmune disease treatment and merits further mechanistic and clinical investigation.

## 6 Therapeutic perspectives

### 6.1 Integrating multi-omics technologies to decipher the gut–immune axis

Recent advances in multi-omics technologies have offered powerful new perspectives for dissecting the complex interplay between the gut microbiota and the host immune system, particularly in the dynamic regulation of Treg and Th17 (159). Integrative applications of metagenomics, metabolomics and transcriptomics show great promise for resolving how microbial communities and their biochemical products influence the differentiation, function and cross-talk of Treg and Th17 (160). For example, SCFAs — key metabolites produced by gut microbes — have been shown to promote the expansion of Treg via receptors such as GPR41 and GPR43 while concurrently restraining the differentiation of Th17, thereby contributing to the maintenance of immune homeostasis (161, 162).

Furthermore, transcriptomic approaches have enabled the identification of specific gene expression profiles and signaling pathways associated with Treg/Th17 balance (163). These studies not only illustrate how microbial metabolites modulate immune responses by altering the host transcriptome but also reveal potential therapeutic targets. For example, certain microbial metabolites can influence the secretion of cytokines such as IL-10 and IL-17, further modulating the Treg/Th17 ratio and thereby affecting the progression of autoimmune diseases (164, 165).

Constructing a cross-tissue regulatory network model of the “gut–immune–target organ” axis is essential for understanding how gut microbiota influence systemic immunity and organ function. Through the integration of multi-omics data, it is possible to map the complex interaction networks among the gut, immune system, and target organs (e.g., liver, lungs) (166). For instance, in chronic inflammatory diseases, gut microbial composition and metabolites can influence the immune status of distant organs via systemic circulation, forming feedback loops along the gut–immune–target organ axis.

Emerging evidence indicates that gut dysbiosis not only triggers local inflammation but may also initiate or exacerbate systemic autoimmune diseases by altering overall immune status. In conditions such as SLE and RA, alterations in gut microbiota and their metabolites are closely associated with disease progression. By establishing cross-tissue regulatory network models, researchers can identify key regulatory factors and their mechanisms, providing a theoretical foundation for precision medicine (167, 168).

### 6.2 Development of personalized microecological therapeutics

The development of individualized microbiome-based therapeutic regimens is a complex but essential undertaking that seeks to design precise interventions by accounting for interindividual variation in gut microbial composition and metabolic output. Studies have shown that the structure of the gut microbiota closely correlates with an individual’s health status, disease susceptibility and therapeutic responsiveness; notably, specific bacterial taxa have been directly linked to the development of metabolic disorders such as obesity and type 2 diabetes (169). Accordingly, tailoring treatments to a patient’s microbiome profile has the potential both to enhance efficacy and to reduce adverse effects.

A first prerequisite for personalized microbiome therapy is the construction of patient-specific gut microbiome databases using high-throughput sequencing to characterize microbial community composition and functional capacity (170, 171). Comparative analyses between healthy subjects and patients enable identification of microbial taxa that are associated with disease states (172). These data can inform predictive models of treatment response and support longitudinal monitoring to permit timely adjustment of therapeutic strategies.

Therapeutic options for microbiome modulation include administration of probiotics, provision of prebiotics, and FMT, among other approaches (173). For example, particular probiotic strains have been reported to increase microbial diversity and bolster immune competence, thereby improving clinical outcomes (174). Dietary interventions likewise exert a demonstrable effect on the intestinal microbiota; specific dietary components can selectively promote the growth of beneficial microbes and thereby contribute to overall health (33).

Finally, successful implementation of individualized microbiome therapies depends on multidisciplinary collaboration among clinicians, microbiologists and nutrition scientists. Integrating mechanistic insights into the “microbiome–metabolite–host” axis will support more finely tuned, patient-centered interventions that enhance therapeutic benefit and improve quality of life for patients (175).

### 6.3 Clinical translation and multicenter large-scale trials

The contributions of the gut microbiota and their metabolites to the study and treatment of autoimmune diseases have attracted growing attention. Recent studies indicate that alterations of the gut microbiota can promote the onset and progression of diverse autoimmune disorders (176). For example, dysbiosis observed in patients with MG is closely associated with an imbalance between Treg and Th17, providing a rationale for microbiota-directed approaches to restore immune equilibrium (177). To facilitate clinical translation of microbiome-based therapies, stronger integration of fundamental research and clinical practice is essential so that mechanistic insights can be reliably converted into effective interventions.

Furthermore, the clinical development of microbiome therapeutics requires well-designed, multicenter, large-scale trials that include geographically and demographically diverse populations to ensure broad applicability and robust evidence. For instance, investigations into postmenopausal osteoporosis have demonstrated that the gut microbiota and its metabolites modulate bone metabolism via the gut–bone axis and the gut–brain axis (178). Systematic evaluation of therapeutic efficacy and safety, together with exploration of applications across different autoimmune conditions, will be critical to inform future clinical practice.

## 7 Limitations and future directions

As our understanding of the gut microbiota and its metabolites deepens, their central role in regulating the balance between Treg and Th17 has become increasingly evident. Treg and Th17 operate as opposing forces within immune networks, and microbial-derived signals that shift their equilibrium provide fresh mechanistic insight into autoimmune disease pathogenesis. A growing body of experimental and clinical evidence shows that restoring a healthy microbial ecology and correcting metabolite profiles can re-establish immune homeostasis and ameliorate disease manifestations across multiple autoimmune disorders. These observations underscore that the integrity of the gut microbiome closely informs systemic immune stability and thereby influences both disease onset and progression.

Building on these mechanistic insights, a range of translational strategies targeting microbial metabolites has emerged. Approaches span direct metabolite supplementation, targeted delivery platforms (including nano-formulations), microbiome-informed personalized regimens, and rational combinations with conventional immunosuppressive agents. Such multidimensional strategies aim to improve therapeutic efficacy while minimizing adverse effects, and individualized treatment programs in particular hold promise for tailoring interventions to patients’ distinct microbiome and immune profiles.

Despite this promise, significant heterogeneity and limitations remain across the literature. Conflicting results often reflect small cohort sizes, divergent study designs, variable analytical pipelines, and limited longitudinal follow-up. These constraints impede causal inference and the identification of robust, generalizable biomarkers. Addressing these gaps will require rigorously powered studies with standardized methods and transparent reporting.

Looking ahead, advancing precision therapies that harness microbial metabolites to recalibrate immune function will depend on tighter integration with clinical practice. Systematic application of multi-omics platforms combined with deep clinical phenotyping can reveal mechanistic links and predictive signatures suitable for clinical translation. Equally important are large-scale, well-controlled clinical trials and long-term follow-up studies to establish safety, efficacy, and durability of microbiome-targeted interventions.

In summary, the gut microbiota and its metabolic products are pivotal determinants of the Treg/Th17 axis and thus of autoimmune disease biology. Modulating microbial communities and their metabolites offers a compelling avenue to restore immune balance and develop innovative treatments. Future efforts that combine multidisciplinary science, rigorous clinical evaluation, and patient-centered design are essential to realize safe, effective, and personalized microbiome-based therapies for autoimmune disease.
